# Association between the *Cytotoxic T-Lymphocyte Antigen 4 *+49G > A polymorphism and cancer risk: a meta-analysis

**DOI:** 10.1186/1471-2407-10-522

**Published:** 2010-10-04

**Authors:** Jian Zheng, Xiao Yu, Lan Jiang, Mang Xiao, Bing Bai, Jiachun Lu, Yifeng Zhou

**Affiliations:** 1 Soochow University Laboratory of Cancer Molecular Genetics, School of Basic Medicine & Biological Sciences, Medical College of Soochow University, Suzhou 215123, China; 2 Department of Otorhinolaryngology-Head and Neck Surgery, Sir Run Run Shaw Hospital, Key Laboratory of Biotherapy of Zhejiang province, Zhejiang University, Hangzhou, China; 3 The Institute for Chemical Carcinogenesis, Guangzhou Medical College, Guangzhou, China

## Abstract

**Background:**

As a key gene in the immunosurveillance of cell malignancy, Cytotoxic T-lymphocyte antigen 4 (CTLA-4 is an important negative regulator of T cell activation and proliferation. The CTLA-4 +49G > A polymorphism is one of the most commonly studied polymorphisms in this gene due to its association with cancer risks, but previous results have been conflicting.

**Methods:**

We preformed a meta-analysis using 22 eligible case-control studies (including 32 datasets) with a total of 11,273 patients and 13,179 controls to summarize the existing data on the association between the *CTLA-4 *+49G > A polymorphism and cancer risk.

**Results:**

Compared with the common *CTLA-4 *+49G > A GG genotype, the carriers of variant genotypes (*CTLA-4 *+49 GC/CC) had a 1.24-fold elevated risk of cancer (95% CI = 1.18-1.32, *P *< 0.05) under the dominant genetic model, as estimated using a fixed effect model. The effect of the *CTLA-4 *+49G > A polymorphism was further evaluated using stratification analysis. In four breast cancer studies, patients with the variant genotypes had a significantly increased risk of breast cancer (OR = 1.31, 95% CI = 1.17-1.48, *P *< 0.00001). A similar result was found in three skin cancer studies (OR = 1.30, 95% CI = 1.10-1.52, *P *= 0.001). In 26 solid tumor studies, subjects with the variant genotypes had a significantly higher risk of developing solid tumors (OR = 1.25, 95% CI = 1.18-1.33, *P *< 0.00001) compared with the 6 non-solid tumor studies (OR = 1.08, 95% CI = 0.79-1.48, *P *= 0.62). Patients with variant genotypes had significantly increased risk of non-epithelial tumors and epithelial tumors, with ORs of 1.23 (95% CI = 1.14-1.32, *P *< 0.00001) and 1.29 (95% CI = 1.17-1.41, *P *< 0.00001), respectively. It was also demonstrated that the increased risk of cancer associated with *CTLA-4 *+49G > A variant genotypes was more pronounced in Caucasians (OR = 1.29, 95% CI = 1.13-1.47, *P *= 0.0002), Asians (OR = 1.23, 95% CI = 1.16-1.32, *P *< 0.00001) and Chinese (OR = 1.23, 95% CI = 1.15-1.31, *P *< 0.00001).

**Conclusion:**

Our meta-analysis suggests that the *CTLA-4 *+49G > A polymorphism genotypes (GA + AA) might be associated with an increased risk of cancer, especially in Caucasians and Chinese.

## Background

The human body generates an immune response to tumors, but it is generally ineffective at causing tumor destruction. One possible reason is that T-cell activation will not occur until two separate signals are received by the cell. The first signal is specific antigen-recognition, which requires T-cell receptors to recognize and bind to major histocompatibility complex (MHC) molecules of antigen-presenting cells (APCs). The second signal is nonspecific and antigen-independent, generated by the interaction between CD28 on the T-cell surface and its ligands CD80 and CD86 on the APCs [1-4]. The CD28 costimulatory pathway plays a critical role in the induction and regulation of autoreactive T-cells. Cytotoxic T-lymphocyte antigen 4 (CTLA-4), a CD28 homologue, is a glycoprotein expressed on activated T-cells that has a high binding affinity with the molecule B7, primarily expressed on APCs [5-8]. Though CTLA-4 shares the same costimulatory ligands as CD28, it delivers a different costimulatory signal. While CTLA-4 is a well documented T-cell negative regulator [3], the underlying molecular basis of its signaling is poorly understood. A large body of evidence supports the hypothesis that CTLA-4 may down-regulate T-cell responses [9-11]. Moreover, the majority of cancer cells either have low immunogenicity, lack costimulatory molecules, or both [12,13]. Therefore, *CTLA-4 *may contribute to NPC development by controlling an individual's immune response.

An A→G dimorphism at position 49 in *CTLA-4 *exon 1 (rs231775) has been reported by Nistico [14], which causes an amino acid change (threonine to alanine) in the peptide leader sequence of the CTLA-4 protein [15]. Recent studies found that this polymorphism may influence the ability of CTLA-4 to bind with B7.1 and subsequently, may affect T-cell activation [16,17]. These results suggest that a G allele instead of an A allele at position +49 can attenuate the CTLA-4-driven down-regulation of T-cell responses [16,18,19]. However, the results of studies on the association between the +49 A > G polymorphism and the risk of cancers have been conflicting. So in order to summarize and clarify the published data we have performed a meta-analysis, using all eligible case-control studies to assess the association between the *CTLA-4 *+49 A > G polymorphism and cancer risk.

## Methods

### Identification and Eligibility of Relevant Studies

We carried out a literature search using the PubMed database (between January 2000 and February 2010) to identify all papers that investigated the association between the *CTLA-4 *+49 A > G polymorphism and cancer risk in all ethnic groups, using combinations of the search phrases "*CTLA-4 *and polymorphism and cancer". We evaluated the titles and abstracts of all relevant publications, but excluded abstracts, case reports, editorials, and review articles. Studies included in the current meta-analysis had to meet the following criteria: the study must have used a case-control study design; the report must have included cancer diagnoses and sources for the cases and controls; the report must have included genotype frequencies; the authors must have given size of their samples, the odds ratios (Ors) used, and their 95% confidence intervals (CIs); definitions of exposure or risk genotypes must have been similar in all reports; and the methods of data collection and analysis must have been statistically acceptable.

### Data extraction and stratification

Data were collected on the CTLA-4 +49 A > G genotype from studies of different types of cancer. The first author, year of publication, country, ethnicity of the study population, the number of cases and controls, and the type of study were described (Table 1). In the stratification analyses for ethnicity, there were: 7 Caucasian populations, including Spanish, Polish, German, American, Sardinian, Macedonian and Italian, and 3 Asian populations, including Chinese (containing Taiwanese), Trukese, and Irani. In the stratification analyses for cancer type, we compared epithelial tumors to non-epithelial tumors, and solid tumors to non-solid tumors. There were 12 types of solid tumors, including those caused by renal cell cancer, colon carcinoma, cervical squamous cell carcinoma, breast cancer, lung cancer, esophageal cancer, gastric cancer, oral squamous cell carcinoma, thymoma, nasopharyngeal carcinoma, HBV-related HCC and melanoma. There were 3 types of non-solid tumors, including those caused by CLL, non-Hodgkin's lymphoma and MALT lymphoma. The 4 types of epithelial tumors were those caused by oral squamous cell carcinoma, cervical squamous cell carcinoma, melanoma, and breast cancer. The 8 non-epithelial tumors included those caused by gastric cancer, colon carcinoma, HBV-related HCC, nasopharyngeal carcinoma, thymoma, renal cell cancer, lung cancer and esophageal cancer.

**Table 1:** Summary of eligible studies considered in the meta-analysis

First author(year)	**Case no**.	**Control no**.	Ethnicity	Country	Cancer type	Matched variables	Type of study
Cozar (2007)	125	176	Caucasian	Spain	Renal Cell Cancer	Age, sex, and residence area matched	Hospital-based
Cozar (2007)	96	176	Spanish	Spain	Colon Carcinoma	Ethnically matched	Hostipal-based
Su (2007)	139	375	Taiwanese Women	China	Cervical Squamous Cell Carcinoma	Age and sex matched	Hostipal-based
Pavkovic (2003)	30	100	Caucasians	Macedonia	CLL (+)AIHA	Age, sex, and residence area matched	Hospital-based
Pavkovic (2003)	100	100	Caucasians	Macedonia	CLL (-)AIHA	Age, sex, and residence area matched	Hospital-based
Suwalska (2008)	170	224	Polish	Poland	B-CLL	Age and sex matched	Hospital-based
Wang (2007)	117	148	Han people	China	Breast Cancer	Age matched	Hospital-based
Bouwhuis (2009)	762	734	German	Germany	Malignant Melanoma	Age matched	Hostipal-based
Sun (2008)	1163	1132	Han people	China	Lung Cancer (Beijing)	Aex matched	Hospital-based
Sun (2008)	1032	1021	Han people	china	Lung Cancer(Jiangsu)	Age-sex and residential area matched	Hospital-based
Sun (2008)	1060	1070	Han people	China	Breast Cancer (Beijing)	Age-sex and residential area matched	Hospital-based
Sun (2008)	1037	1070	Han people	China	Breast Cancer (Jiangsu)	Age-sex and residential area matched	Hospital-based
Sun (2008)	1010	1008	Han people	China	Esophagus Cancer (Beijing)	Age-sex and residential area matched	Hospital-based
Sun (2008)	530	530	Han people	China	Gastric Cardia Cancer (Beijing)	Age-sex and residential area matched	Hospital-based
Hou (2010)	205	262	Han people	China	Gastric Cancer		
Dilmec (2008)	56	162	Trukese	Turkey	Colorectal Cancer	Age, sex and ethnically matched	Hostipal-based
Welsh (2009)	897	819	New Hampshire people	American	Non-melanoma Skin Cancer BCC	Age and sex matched	Hospital-based
Welsh (2009)	684	819	New Hampshire People	American	Non-melanoma Skin Cancer SCC	Age, sex, and residence area matched	Hospital-based
Hadinia (2007)	105	190	Irani	Iran	Colorectal Cancer		Hostipal-based
Hadinia (2007)	43	190	Irani	Iran	Gastric Cancer	Age and sex matched	Hostipal-based
Wong (2006)	118	147	Taiwan People	China	Oral Squamous Cell Carcinoma	Age and sex matched	Hostipal-based
Piras (2005)	100	128	Sardinia People	Sardinia	Non-Hodgkin's Lymphoma	Sex matched	
Ghaderi (2004)	197	151	Iranian Women	Iran	Breast Cancer	Age matched	Hostipal-based
Qi (2010)	124	407	Chinese	China	Colorectal Cancer	Age and sex matched	
Cheng (2006)	62	250	Han Chinese	China	MALT Lymphoma	Age and sex matched	
Solerio (2005)	186	238	Caucasian	Italian	Colorectal Adenomas	Age, sex, and residence area matched	Hospital-based
Solerio (2005)	132	238	Caucasian	Italian	Colorectal Cancers	Age and sex matched	
Monne (2004)	44	76	Caucasian	Italy	Non-Hodgkin's Lymphomas(NHL)	Age and sex matched	Hospital-based
Chuang (2005)	79	173	White Germans	Germany	Myasthenia gravis MG(+)Thymoma		Hospital-based
Chuang (2005)	46	173	White Germans	Germany	Myasthenia gravis MG(-)Thymoma		Hospital-based
Xiao (2009)	457	485	Han Chinese	China	Nasopharyngeal Carcinoma	Age and sex matched	Hospital-based
Gu (2010)	367	407	Han Chinese	China	HBV-related HCC		Hospital-based

### Methods for quantitative synthesis

The selection of published studies we used for meta-analysis were further evaluated using sensitivity analyses. Odds ratios (ORs) and 95% confident interval (CIs) from each case-control study were used to assess the strength of association between the *CTLA-4 *+49A > G genotypes and the risk of cancer in dominant (GA+AA *vs *GG) genetic models. A combined OR was calculated according to Woolf's method [20]. A χ2-based Q statistic test was performed to assess between-study heterogeneity [21]. If the *P *value of the heterogeneity test was ≥ 0.05, then a fixed effect model using the Mantel-Haenszel method was used to calculate the combined OR, which assumed the same homogeneity of effect size across all studies. If the *P *value of the heterogeneity test was <0.05, it showed that the between-study heterogeneity was statistically significant. A random effects model, using the DerSimonian and Laird method, was performed to calculate the combined OR [22]. If there were no between-study heterogeneity, the combined OR calculated by those two methods would be identical. The significance of the combined OR was determined using a *Z*-test, in which *P *< 0.05 was considered significant. Finally, the combined ORs and their 95% CIs were presented. Stratification analyses for different types of cancers were conducted for colorectal cancer, breast cancer, gastric cancer, lymphoma, skin cancer, and other cancers (lung cancer, nasopharyngeal carcinoma, cervical squamous cell carcinoma, esophageal cancer, oral squamous cell carcinoma, HBV-related hepatocellular carcinoma or renal cell cancer) to estimate cancer-specific ORs. Stratification analyses by ethnicity were also conducted for Caucasian, Chinese and other Asian race populations to estimate ethnicity-specific ORs. Publication bias was assessed using a funnel plot, in which the standard error of log (OR) of each study was plotted against its OR value. The resulting asymmetrical plot suggested, according to Egger's linear regression test, that there was possible publication bias [23]. The significance of the intercept was determined using a Student *t*-test, as suggested by Egger. If the *P*-value of Egger's linear regression test was less than 0.05, it meant that there was a publication bias in the meta-analysis. The SAS/Genetics software program (Version 9.1, SAS Institute, Inc., Cary, NC, USA) was used to determine the LD of SNP pairs and the Hardy-Weinberg equilibrium. Other statistical software used included SPSS12.0 for Windows (SPSS Inc., Chicago, USA), Stata Version 10.0, and Review Manager (Version 4.2, the Cochrane Collaboration). All *P*-values were two-sided.

## Results

### Literature search and meta-analysis databases

We searched NCBI PubMed using the key terms '*CTLA-4*' 'cancer' and 'polymorphism' and found 36 epidemiological studies. Of these 36 studies, 14 studies were excluded either because they were not case-control studies or because the +49A/G polymorphism or its genotype frequency was not reported. The remaining 22 case-control studies contained 32 data sets (Sun's study had six datasets, and Pavkovic's, Welsh's, Hadinia's, Solerio's, and Chuang's studies all had two datasets each) [16,24-28]. We created a database of the information extracted from each article. The essential information, including first author, cancer type, year of publication, numbers of cases and controls, and the frequencies of CTLA-4 +49A/G for all studies are listed in Table 1. There were four studies concerning breast cancer [16,17,29], six concerning colorectal cancer [25,27,30-32], six concerning non-solid tumors [26,33-36], three concerning gastric cancer [16,25,37], three concerning skin cancer [28,38], two concerning Thymoma [24], two concerning lung cancer [16], one concerning nasopharyngeal carcinoma [39], one concerning cervical squamous cell carcinoma [40], one concerning Esophagus cancer [16], one concerning Oral squamous cell carcinoma [41], one concerning HBV-related hepatocellular carcinoma [42], and one concerning renal cell cancer [30]. Among the 32 datasets included in the final analysis, there were 14 concerning Caucasians and 18 concerning Asians (14 concerning Chinese, one concerning Trukese and three concerning Irani). Additional information is listed in the forest plots in our meta-analyses. We performed a sensitivity analysis on the selection of published studies in this meta-analysis.

### Test for heterogeneity

**Figure 1:**
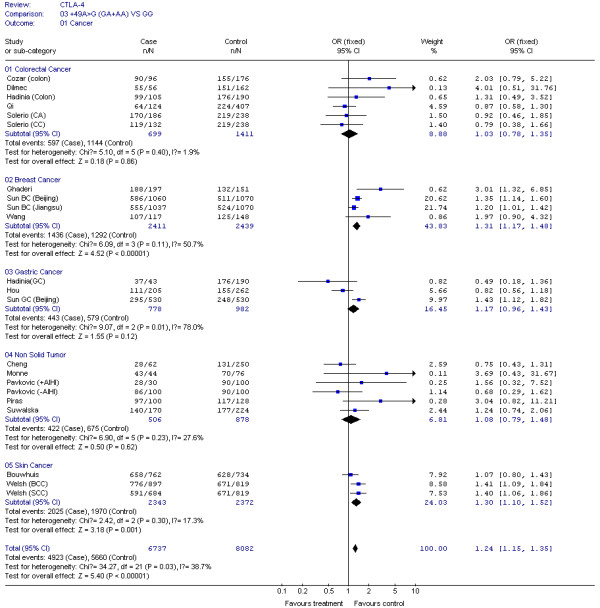
**Meta-analysis for CTLA-4 +49G > A polymorphism variant genotypes GA + AA *vs*. GG in different type of cancers**.

**Figure 2:**
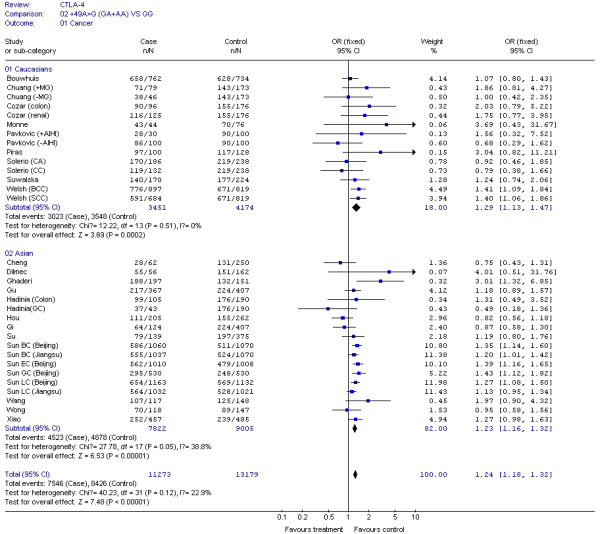
**Meta-analysis for CTLA-4 CTLA-4 +49G > A polymorphism variant genotypes GA + AA *vs*. GG in different ethnicities**.

**Figure 3:**
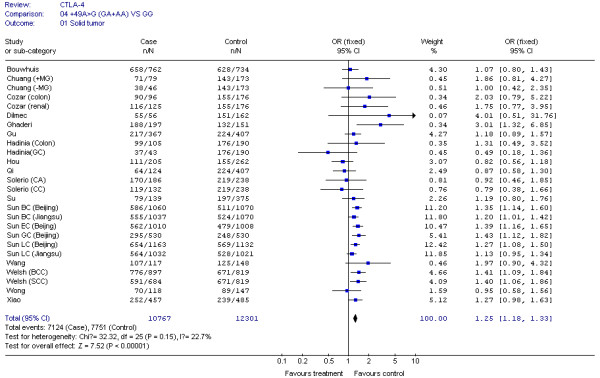
**CTLA-4 +49G > A polymorphism variant genotypes GA + AA *vs*. GG in Solid tumor**.

**Figure 4:**
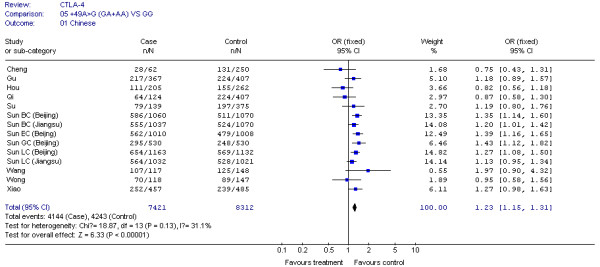
**CTLA-4 +49G > A polymorphism variant genotypes GA + AA *vs*. GG in Chinese**.

**Figure 5:**
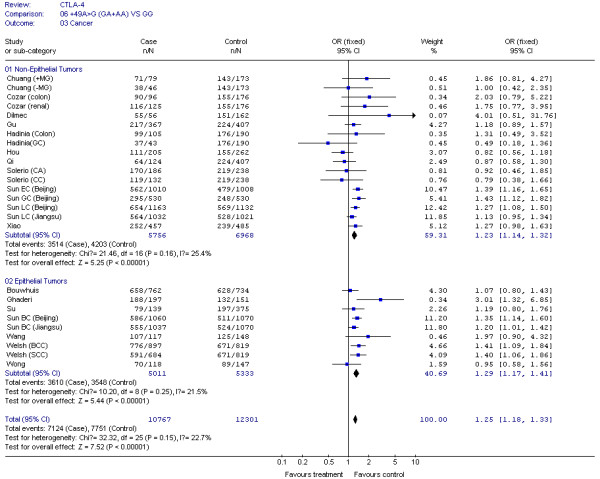
**CTLA-4 +49G > A polymorphism variant genotypes GA + AA *vs*. GG in epithelial and non-epithelial tumors**.

The results of our Q test show that there was no between-study heterogeneity in the dominant genetic models (GA+AA *VS *GG) for all 32 datasets (*P *= 0.12). In the subgroup analyses for cancer type and ethnicity (results shown in Figures 1, 2, 3, 4 and 5), the heterogeneity test did not reveal any significant differences between the dominant genetic models (GA+AA *VS *GG): six colorectal cancer studies (*P *= 0.40), four breast cancer studies (*P *= 0.11), six non-solid tumor studies (*P *= 0.23), three skin cancer studies (*P *= 0.30) (Figure 1), and twenty-six solid tumor studies (*P = *0.15) (Figure 3), seventeen non-epithelial tumor studies (*P *= 0.16), nine epithelial tumor studies (*P *= 0.25) (Figure 5), fourteen Caucasian population studies (*P *= 0.51), fourteen Chinese population studies (*P *= 0.13), and eighteen Asian studies (*P *= 0.05) (Figure 2 and 4). However, the results of our heterogeneity test did indicate significant differences (*P *= 0.01) for gastric cancer (Figure 1).

### Quantitative data synthesis

**Figure 6:**
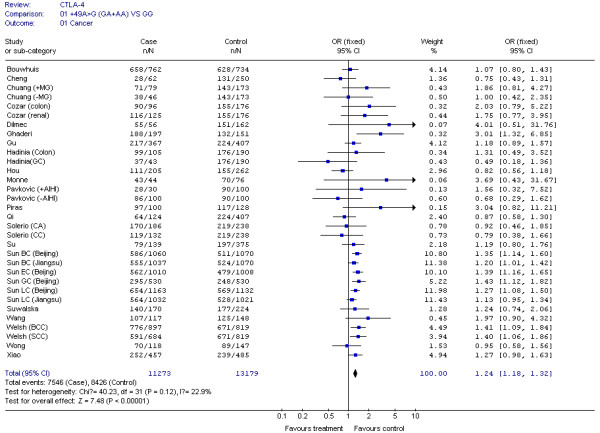
**CTLA-4 +49G > A polymorphism variant genotypes GA + AA *vs*. GG in all cancers**.

For the CTLA-4 +49G > A polymorphism, we obtained our meta-analysis data from 32 datasets consisting of 11,273 cases and 13,179 controls. The associations between the CTLA-4 +49G > A genotype and cancer risks were estimated using dominant (GA+AA *vs *GG) genetic models in either fixed or random effect models according to the heterogeneity Q test. We used 32 datasets in these comparisons. Compared with the wild-type +49G > A GG genotype, the carriers of variant genotypes (GA/AA) had a 1.24-fold elevated risk of cancer (95% CI = 1.18-1.32, *P <*0.05) as estimated by a fixed effect model for dominant genetic effects (Figure 6).

The effect of the CTLA-4 +49G > A polymorphism was further evaluated using stratification analysis. In the six colorectal cancer studies, which included 699 cases and 1,411 controls, subjects with variant genotypes (597 cases and 1,144 controls) had a non-significant increased risk of colorectal cancer (OR = 1.03, 95% CI = 0.78-1.35, *P *= 0.86) as estimated using a fixed effect model (Figure 1). Similar results were found in the three gastric cancer studies, with 778 cases and 982 controls (OR = 1.17, 95% CI = 0.96-1.43, *P *= 0.12), and the six non solid tumour studies, with 506 cases and 878 controls (OR = 1.08, 95% CI = 0.79-1.48, *P *= 0.62) (Figure 1). However, in the four breast cancer studies, consisting of 2,411 cases and 2,439 controls, the variant genotypes (1,436 cases and 1,292 controls) were associated with a significantly increased risk of breast cancer (OR = 1.31, 95% CI = 1.17-1.48, *P *< 0.00001) (Figure 1). Similar results were found in the three skin cancer studies, which included 2,342 cases and 2,372 controls (OR = 1.30, 95% CI = 1.10-1.52, *P *= 0.001), and in the 26 solid tumour studies, which had 10,767 cases and 12,301 controls (OR = 1.25, 95% CI = 1.18-1.33, *P *< 0.00001) (Figure 1 and 3). We also found that patients with the variant genotypes had significantly increased risks for developing either non-epithelial tumors or epithelial tumors, with ORs of 1.23 (95% CI = 1.14-1.32, *P *< 0.00001) and 1.29 (95% CI = 1.17-1.41, *P *< 0.00001), respectively (Figure 5).

In the stratification analyses for ethnicity, we found that the increased risk of cancer associated with +49G > A variant genotypes was more pronounced in Caucasians (OR = 1.29, 95% CI = 1.13-1.47, *P *= 0.0002), Asians (OR = 1.23, 95% CI = 1.16-1.32, *P *< 0.00001), and Chinese (OR = 1.23, 95% CI = 1.15-1.31, *P *< 0.00001) (Figure 2 and 4).

### Bias diagnostics

**Figure 7:**
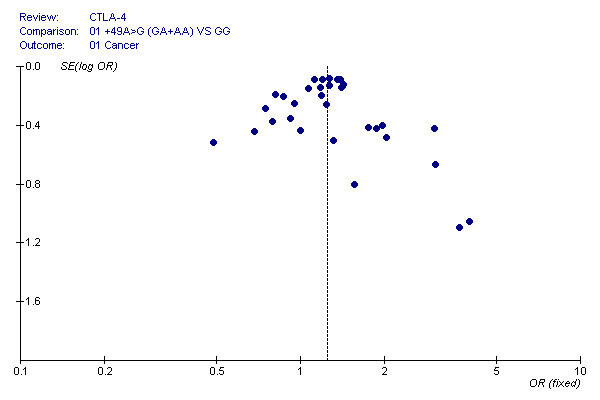
**Funnel plot of the Egger's test of CTLA-4 +49G > A polymorphism for publication bias**.

To evaluate publication bias, the CTLA-4 +49G > A genotypes were plotted against the precision ones using a funnel plot. The result was approximately symmetrical, so Egger's test suggests that there is no publication bias in the current meta-analysis (*P *= 0.998). This indicates that biases from publications and other factors may not have had a significant influence on the results of our meta-analysis on the association between CTLA-4 +49G > A polymorphism and cancer risk (Figure 7).

## Discussion and Conclusions

In this meta-analysis, which includes 22 independent case-control studies with 32 data sets, we found that the carriers of the CTLA-4 +49 (GA+AA) variant genotypes had a 1.24-fold increased risk of cancer in the dominant genetic model. These results support the hypothesis that polymorphisms of CTLA-4 play an important role in the development of cancer. However, we did not find evidence of significant associations in subgroup analyses for individual types of cancers, such as colorectal cancer, gastric cancer and non-solid tumors.

The immune system is a complex network that has evolved to protect humans against infectious agents and tumor growth. T-cells and natural killer (NK) cells are the major anti-tumor factors. Given that the activation of T-cells requires two signals, the CD28 costimulatory pathway has been shown to play a critical role in the induction and regulation of autoreactive T-cells. Furthermore, the human *CTLA4 *and *CD28 *genes are located in the same chromosome region and are closely linked and separated by only 130 kb [43]. The gene structure of *CTLA4 *is very similar to that of *CD28*, except for 3' and 5' flanking sequences. All these data suggest that CTLA-4 and CD28 may be members of the same pathway, but execute different functions. Several groups found that CTLA-4 binds to the same ligands as CD28, i.e. CD80 and CD86 molecules, but has at least a 20-fold greater affinity [44]. In contrast to CD28, CTLA-4 does not provide a positive signal for T-cell activation. Thus, CTLA-4 may contribute to the down-regulation of anti-tumor immune responses via interference with the CD28 costimulatory pathway. Moreover, CTLA-4 has been reported to increase TGF production, engage negative signaling pathways, inhibit lipid-raft and disturb TCR-induced stop signals [11,45,46]. Nistico and his colleagues reported a functional polymorphism in *CTLA4 *exon 1 which causes a threonine to alanine amino acid exchange in this protein's leader sequence [14]. We noted the impact of the *CTLA4 *exon 1 + 49 A/G dimorphism on immune regulation after T-cell stimulation. Several studies found that the surface expression and intracellular distribution of CTLA-4 differ between the two genotypes [18,19]. Results of the T-cell proliferation and B7.1 binding capability studies by Sun *et al*. suggest that the *G *allele at this position is correlated with increased T-cell activation [16].

This study evaluated the associations of CTLA-4 +49A/G polymorphisms with different cancers. We found that this polymorphism was associated with an increased risk of developing solid tumors (including lung caner, breast cancer, colorectal cancer, gastric cancer, skin cancer, thymoma, nasopharyngeal carcinoma, cervical squamous cell carcinoma, esophageal cancer, oral squamous cell carcinoma, HBV-related hepatocellular carcinoma, and renal cell cancer), but not non-solid tumors, suggesting that the CTLA-4 gene plays different roles in the carcinogenesis of these two types of tumors. In our stratified analysis for ethnicity, the *CTLA-4 *+49G > A variant genotypes (GA + AA) were associated with an increased risk of cancer in Caucasians (OR = 1.29, 95% CI = 1.13-1.47, *P *= 0.0002), Chinese (OR = 1.23, 95% CI = 1.15-1.31, *P *< 0.00001), and Asians (OR = 1.23, 95% CI = 1.16-1.32, *P *< 0.00001), suggesting that the different genetic backgrounds of the different populations may to some extent explain the different risk estimates associated with the variant CTLA-4 genotypes. It seems that certain populations may have a higher susceptibility to cancer because they have higher frequencies of the variant genotypes +49G > A (GA + AA). Potential publication biases may exist in this meta-analysis, because studies with negative results are less likely to be published. Because only four out of 32 datasets were population-based case-control studies, with the others being hospital-based case-control studies, the study subjects may also not be representative of the general population. This could lead to selection bias.

In conclusion, our meta-analysis found evidence for an association between CTLA-4 +49A/G polymorphisms and multiple cancers in the general population, particularly for solid tumors. Due to the limitations of meta-analyses, larger association studies or multi-centric case-control studies are needed to confirm these findings.

## Competing interests

The authors declare that they have no competing interests.

## Authors' contributions

JZ and XY participated study design and drafted the manuscript. JL carried out bioinformatics analysis and critically revised the manuscript. LJ and MX performed the statistical analysis. BB participated in the critical revision of the manuscript. YZ conceived of the study, and participated in its design and coordination. All authors read and approved the final manuscript.

## Pre-publication history

The pre-publication history for this paper can be accessed here:

http://www.biomedcentral.com/1471-2407/10/522/prepub
